# Associations Between Type D Personality, Psychological Symptoms, Pain Severity, and Migraine-Related Outcomes in Women with Migraine

**DOI:** 10.3390/jcm15135048

**Published:** 2026-06-29

**Authors:** Meltem Hazel Şimşek, Hüsniye Aylin Dikbaş, Ulaş Korkmaz

**Affiliations:** 1 Department of Psychiatry, Faculty of Medicine, Giresun University, Giresun 28200, Türkiye; 2 Department of Neurology, Faculty of Medicine, Giresun University, Giresun 28200, Türkiye

**Keywords:** Type D personality, migraine, headache impact, functional impairment

## Abstract

**Background/Objectives:** Migraine is common in women and is associated with significant disability and reduced quality of life. Type D personality, characterized by negative affectivity and social inhibition, has been linked to poorer outcomes in chronic illnesses; however, its association with migraine-related impact and functioning in women has not been fully clarified. This study examined the associations of Type D personality with headache impact and overall functioning in women with migraine, with an exploratory focus on the indirect statistical associations involving psychological symptoms, pain severity, and age. **Methods:** In this cross-sectional study, 204 women with migraine were assessed. Type D personality was measured using the DS14, headache impact using the HIT-6, and functioning using the FAST. Anxiety and depression were measured with the HADS, and pain severity with a visual analog scale. Analyses included group comparisons, correlations, exploratory mediation analyses, and moderation analyses. **Results:** Type D personality was present in 41.2% of participants. Women with Type D personality had more chronic migraine, physical comorbidities, lower education and employment, and greater functional impairment. Type D personality was correlated with headache impact, functional impairment, anxiety, and depression. Anxiety and pain severity showed significant indirect statistical associations in the relationship between Type D personality and headache impact, while depression showed a significant indirect statistical association in the relationship between Type D personality and functioning. Age moderated the association between Type D personality and headache impact, with a stronger association observed in older women. **Conclusions:** In women with migraine, Type D personality was associated with greater functional impairment, whereas its association with headache impact was attenuated after adjustment for relevant clinical and psychological factors. Psychological symptoms and pain severity showed significant indirect statistical associations with migraine-related outcomes; however, causal inferences cannot be drawn due to the cross-sectional design. These findings highlight the potential clinical relevance of assessing personality traits and psychological symptoms in women with migraine.

## 1. Introduction

Migraine is a neurovascular disorder affecting approximately 15% of the population and ranks as the second leading cause of disability worldwide [1]. As with many chronic conditions, migraine is more prevalent among women than men [1,2,3,4,5]. Given the well-established sex-related differences in migraine prevalence, clinical presentation, and disease burden, focusing specifically on women may provide a more homogeneous framework for investigating migraine-related outcomes. In women, migraine tends to be more severe, longer-lasting, and more frequent. It is also associated with greater functional impairment and poorer quality of life [6,7]. It has been shown that an important contributor to this sex-related difference is fluctuations in hormone levels, such as estrogen and progesterone [6]. Moreover, with menopause, the reduction in hormonal fluctuations and the increase in hormonal stability may be associated with a decrease in migraine severity and its overall impact [8].

Based on the duration of migraine symptoms within a one-month period, migraine is classified as episodic or chronic migraine [9]. Chronic migraine is associated with greater functional impairment, higher disease burden, and poorer quality of life compared with episodic migraine [10]. Psychiatric comorbidities are an important risk factor in the chronification of migraine [10,11]. On the other hand, elevated levels of anxiety and depression may contribute to a more severe experience of migraine [10]. The prevalence of anxiety among patients with migraine is two to four times higher than in the general population [12]. Previous studies have shown that approximately 25% of patients with migraine exhibit clinically significant depressive symptoms [13]. The presence of psychiatric comorbidities accompanying migraine has been associated with increased frequency and severity of the disorder, marked reductions in functioning, and poorer quality of life [14]. In a large-scale study, the presence of comorbid depression was found to increase the risk of migraine-related disability by 56%, while the co-occurrence of anxiety and depression raised this risk to 79% [15]. This finding points to a multidimensional and complex relationship between migraine and mental disorders.

Migraine frequency and severity may be influenced by certain personality traits and vulnerability to stress. In recent years, particular attention has been given to personality characteristics that may be associated with migraine. One such trait, Type D personality (TDP), is defined as a distressed personality characterized by negative affectivity and social inhibition [16]. It has been reported that the prevalence of TDP among individuals diagnosed with migraine is 45.2% and is significantly higher than that observed in the general population [16,17]. Several studies have demonstrated that individuals with migraine who exhibit TDP traits experience more frequent attacks, greater headache severity, and more pronounced functional impairment [16,18]. In addition, personality-related psychopathologies such as depression and anxiety are more frequently observed in patients with migraine [19]. However, the relationship between TDP and migraine in women has not been sufficiently investigated.

Given that migraine substantially impairs functioning and that co-occurring Type D personality may be associated with poorer functional outcomes, investigating these relationships appears particularly important. However, studies investigating the associations among Type D personality, psychological symptoms, headache impact, and functioning in women with migraine remain limited. To the best of our knowledge, no previous study has specifically focused on women with migraine while simultaneously examining the relationships among Type D personality traits, anxiety, depression, pain severity, headache impact, and functional impairment. Considering the higher prevalence and greater burden of migraine among women, a detailed evaluation of these associations is of particular clinical relevance. Exploring potential indirect statistical associations among these variables may contribute to a more comprehensive understanding of the biopsychosocial profile of women with migraine. Because of the cross-sectional design, these indirect associations were considered exploratory and were not intended to establish temporal or causal pathways.

In this study, we aimed to examine the associations between Type D personality traits, psychological symptoms, headache impact, and functioning in women diagnosed with migraine, as well as exploratory indirect statistical associations among these variables.

The study hypotheses were as follows:

**H1.**  *Women with migraine who exhibit TDP traits experience a greater negative impact of headache on daily functioning and have lower overall functioning levels compared with those without TDP traits.*

**H2.**  *In women with migraine, anxiety and depression levels are negatively associated with headache-related daily functioning and overall functioning.*

**H3.**  *In women with migraine, anxiety, depression, and pain severity are expected to show significant indirect statistical associations in the relationship between Type D personality traits and both headache impact and overall functioning.*

**H4.**  *Age moderates the relationship between TDP traits and the impact of headache on daily life.*

## 2. Materials and Methods

### 2.1. Study Design and Sample

The study included 204 women diagnosed with migraine who presented to the neurology outpatient clinic of a tertiary care hospital between April and November 2025. Eligible patients presenting to the neurology outpatient clinic during the study period were recruited consecutively until the target sample size was reached. The diagnosis of migraine was confirmed by a neurologist through face-to-face clinical evaluation and review of medical records, in accordance with the criteria of the International Classification of Headache Disorders, 3rd edition (ICHD-3) [20]. Only women were included in the study to reduce sex-related heterogeneity and because migraine is substantially more prevalent and clinically burdensome among women. In addition, participants were evaluated by a psychiatrist with respect to psychiatric diagnoses, based on medical records and clinical interviews.

This study was approved by the Ethics Committee of Giresun Training and Research Hospital (Decision No: 09.04.2025/12; Date: 9 April 2025). Written informed consent was obtained from all participants. The study was conducted in accordance with the principles of the Declaration of Helsinki.

The exclusion criteria of the study included the presence of tension-type headache, trigeminal autonomic cephalalgias, or other primary headache disorders, as well as secondary causes of headache that could mimic migraine. Neurological comorbidities such as epilepsy, a history of stroke, and demyelinating diseases were also grounds for exclusion. In addition, individuals with psychotic disorders, bipolar disorder, substance use disorders, or severe psychiatric conditions that could interfere with the reliable completion of the assessment scales were excluded. Furthermore, participants with active systemic infections, malignancy, advanced cardiac, hepatic, or renal failure, high-dose systemic corticosteroid use within the previous month, pregnancy or breastfeeding, cognitive impairment, or insufficient literacy to complete the questionnaires were not included in the study. Patients receiving migraine prophylactic treatment at the time of assessment were not included in the study.

During the recruitment period, 256 patients were screened. Of the 52 excluded participants, 14 were receiving prophylactic migraine medication, 11 had psychiatric disorders meeting exclusion criteria, 8 had secondary headache disorders, 7 had insufficient literacy to complete the questionnaires, and 12 were excluded for other predefined reasons.

### 2.2. Data Collection Instruments

Sociodemographic and Clinical Data Form: This form included basic sociodemographic information such as age, gender, and occupation. It also collected data on migraine duration, migraine attack frequency, number of headache days, migraine type, smoking pack-years, comorbid medical conditions, and medications used. Headache severity was assessed using a 0–10 Visual Analog Scale (VAS), where 0 indicated “no pain”, and 10 indicated “unbearable pain.” The form was completed by the physician during face-to-face interviews with the patients.

Type D Personality Scale (DS14): This scale was developed by Denollet [21] and is used to assess levels of negative affectivity and social inhibition. It consists of two subscales, each comprising seven items, for a total of 14 items. Participants rate each item on a 5-point Likert-type scale. A score of ≥10 on each subscale indicates the presence of TDP traits. The study on the validity and reliability of the Turkish version was conducted by Öncü et al. [22]. In the scale, Cronbach’s alpha coefficients were 0.85 for negative affectivity and 0.76 for social inhibition. In the current sample, Cronbach’s alpha coefficients were 0.901 for negative affectivity and 0.71 for social inhibition.

Hospital Anxiety and Depression Scale (HADS): This self-report scale was developed by Zigmond and Snaith [23] to assess symptoms of anxiety and depression. It consists of a total of 14 items, with seven items evaluating anxiety (HADS-A) and seven items assessing depression (HADS-D). Each item is rated on a 0–3 scale, yielding subscale scores ranging from 0 to 21. The Turkish validity and reliability study was conducted by Aydemir et al. [24]. In the present study, Cronbach’s alpha coefficients were 0.8525 for the anxiety subscale and 0.7784 for the depression subscale.

Headache Impact Test–6 (HIT-6): This scale was developed by Ware et al. [25] to assess the impact of headache on daily life. It is a six-item measure that evaluates the effects of headache on pain severity, functioning, social and physical activities, and concentration. Items are rated on a five-point frequency scale. Each item is scored between 6 and 13, resulting in a total score ranging from 36 to 78. Higher total scores indicate a greater negative impact of headache on an individual’s life. The Turkish validity and reliability study of the scale was conducted by Dikmen et al. [26], with a reported Cronbach’s alpha internal consistency coefficient of 0.89. In the current sample, the Cronbach’s alpha coefficient was 0.873.

Functioning Assessment Short Test (FAST): This scale was developed by Rosa et al. [27] to assess functioning. It consists of 24 items rated on a four-point Likert-type scale ranging from 0 to 3. The scale evaluates functioning across six domains: autonomy, occupational functioning, cognitive functioning, financial issues, interpersonal relationships, and leisure activities. In addition, the total score derived from all items is used to determine overall functioning. Total scores range from 0 to 72, with higher scores indicating greater functional impairment. The Turkish validity and reliability study of the scale was conducted by Aydemir et al. [28], and a Cronbach’s alpha coefficient of 0.96 was reported. In the current sample, the Cronbach’s alpha coefficient was 0.893.

### 2.3. Statistical Analysis

Statistical analyses were performed using IBM SPSS Statistics version 27. The normality of continuous variables was assessed by examining whether skewness and kurtosis values fell within ±1.5 [29]. Continuous variables with a normal distribution were reported as mean ± standard deviation, whereas non-normally distributed variables were reported as median (first quartile–third quartile). Categorical variables were expressed as frequencies and percentages (%).

In the statistical analyses, Type D personality was evaluated as a categorical variable (Type D+ vs. Type D−) for all group comparisons (e.g., *t*-tests, Mann–Whitney U tests, chi-square tests, and MANCOVA). Conversely, the DS14 total score and its subscales were utilized as continuous variables for correlation, mediation, and moderation analyses. Comparisons between the Type D personality–positive (Type D+) and Type D personality–negative (Type D−) groups were conducted using independent samples *t*-tests when appropriate, and Mann–Whitney U tests when parametric assumptions were not met. Categorical variables between the two groups were analyzed using the chi-square test. To control for the effects of variables that differed significantly between groups, analyses of covariance were performed. In particular, group differences in HIT-6 scores, FAST total scores, and FAST subscale scores were evaluated using multivariate analysis of covariance (MANCOVA).

Anxiety and depression scores were included as covariates in the MANCOVA models to examine whether the associations between Type D personality and outcome measures remained after accounting for concurrent psychological symptom levels. In contrast, these variables were examined as potential mediators in separate exploratory PROCESS analyses to evaluate their possible indirect statistical associations with the observed relationships. Thus, the covariate-adjusted analyses and mediation analyses were designed to address different research questions.

The relationships between TDP and other variables were examined using Pearson correlation analysis. Spearman correlation analysis was applied when the assumption of normal distribution was not met.

To explore potential indirect statistical associations between TDP and outcome variables, exploratory mediation analyses were conducted. Because all variables were measured at the same time point, these analyses were not intended to establish temporal ordering among variables. In these analyses, Hayes’ PROCESS macro (Model 4) was used to examine the indirect associations of HADS-Anxiety, HADS-Depression, and VAS in the relationships between DS14 scores and both HIT-6 and FAST scores [30]. Using Model 4, total, direct, and indirect association coefficients were calculated. The significance of indirect associations was assessed using the bootstrapping method with 5000 resamples to generate 95% confidence intervals. The magnitude of the indirect association was reported using the kappa-squared (K^2^) statistic. K^2^ was reported as an effect size measure for these associations, representing the ratio of the observed indirect association to the maximum possible indirect association given the constraints of the data. An effect size is interpreted as a small effect if it is close to 0.01, a medium effect if it is close to 0.09, and a large effect if it is close to 0.25 [31]. Given the cross-sectional design of the study, these mediation analyses were interpreted as exploratory statistical models of indirect associations rather than evidence of temporal or causal pathways.

In addition, moderation analyses were conducted to examine whether the associations between TDP and outcome variables differed across specific subgroups. In particular, the moderating role of age in the relationship between TDP and headache impact was tested using the PROCESS macro (Model 1), and the interaction effect was illustrated graphically.

Given the exploratory nature of the correlation, mediation, and moderation analyses, no formal correction for multiple comparisons was applied. Therefore, these findings should be interpreted with appropriate caution. For all analyses, the level of statistical significance was set at *p* < 0.05.

### 2.4. Sample Size and Power Analysis

A post hoc power analysis was conducted using GPower software (version 3.1) to evaluate the statistical power of the adjusted group comparisons. Assuming a medium effect size (f = 0.25), an alpha level of 0.05, and a total sample size of 204, the achieved power was calculated to be 0.944. Furthermore, regarding the mediation models, Fritz and MacKinnon [32] established that a sample size of approximately 71 to 115 is sufficient to achieve 0.80 power for detecting medium-sized mediated effects using bias-corrected bootstrapping. Therefore, our sample size of 204 was deemed adequately powered for both the primary adjusted comparisons and the mediation analyses.

## 3. Results

A total of 204 women with migraine were included in the study, of whom 84 (41.2%) were identified as having TDP traits. The basic sociodemographic characteristics of the Type D+ and Type D− groups are presented in Table 1. No statistically significant differences were observed between the two groups in terms of mean age, marital status, place of residence, smoking and alcohol use, or rates of regular physical activity. In contrast, the mean duration of education was significantly lower in the Type D+ group (*p* = 0.004). In addition, the proportion of unemployed participants was significantly higher in the Type D+ group compared with the Type D− group (*p* < 0.001) (Table 1).

Comparisons of clinical characteristics, psychiatric symptoms, and functioning levels according to the presence of TDP are presented in Table 2. The prevalence of chronic migraine was higher among Type D+ patients (*p* = 0.018). The two groups were similar with respect to a history of psychiatric illness, with no statistically significant difference observed (*p* = 0.261). In addition, the presence of chronic medical comorbidities was more frequent in the Type D+ group compared with the Type D− group (*p* = 0.032). Type D+ patients also exhibited a higher monthly frequency of migraine attacks (number of headache days per month; *p* = 0.014). In contrast, no significant between-group differences were found in migraine duration (years), mean attack duration (minutes), or mean pain severity (VAS). Anxiety and depression levels were significantly higher in the Type D+ group than in the Type D− group (both subscales, *p* < 0.001). Group comparisons of HIT-6 and FAST scores were conducted using multivariate analysis of covariance (MANCOVA). Education level, employment status, migraine type, presence of chronic medical comorbidity, number of headache days per month, and anxiety and depression scores were included as covariates. Although HIT-6 scores were higher in the Type D+ group (66.3 ± 6.0 vs. 62.7 ± 8.5), this difference did not reach statistical significance (*p* = 0.171). In contrast, the FAST total score was significantly higher in the Type D+ group (*p* = 0.005). Among FAST subscales, autonomy, cognitive functioning, financial issues, and interpersonal relationships scores were significantly higher in the Type D+ group compared with the Type D− group (all *p* < 0.05). However, no significant differences were observed between the groups in occupational functioning or leisure activities subscale scores. When covariates were not controlled for, HIT-6 (F = 11.564, *p* < 0.001), occupational functioning (F = 32.552, *p* < 0.001), and leisure activities (F = 20.413, *p* < 0.001) scores were higher in the Type D+ group (Table 2). Additionally, because anxiety and depression were examined as potential mediators in separate exploratory analyses, additional models excluding these variables as covariates were also examined. Under these conditions, the difference in HIT-6 scores remained non-significant (F = 0.263, *p* = 0.609), whereas all functioning domains remained statistically significant (*p* < 0.005).

To examine the linear relationships, the continuous DS14 total and subscale scores were used. Correlations between TDP and clinical and psychosocial variables are presented in Table 3. DS14 total scores and its Negative Affectivity and Social Inhibition subscales showed significant positive correlations with anxiety levels, depression levels, the impact of headache on quality of life, and measures of functioning (all *p* < 0.001). In addition, pain severity scores and the number of headache days per month also exhibited statistically significant but weaker positive correlations with HIT-6 and certain FAST subscale scores (*p* < 0.05). Higher HADS-A and HADS-D scores were likewise significantly associated with increased HIT-6 and FAST scores (*p* < 0.001) (Table 3).

**Figure 1:**
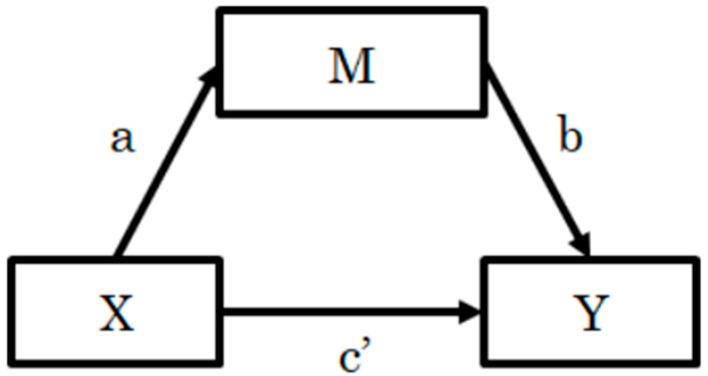
PROCESS Macro model 4.

The statistically indirect associations involving anxiety, depression, and pain severity in the relationships between DS14 scores and both HIT-6 and FAST scores were evaluated using Hayes’ PROCESS Model 4 (Table 4). In these analyses, DS14 score was used as the independent variable (X), and HIT 6 and FAST scores were used as the dependent variables (Y). HADS-A score, HADS-D score, and VAS score were included in the model as mediators (M). Figure 1 illustrates the proposed model. The “a” path represents the association of the independent variable (X) with the mediator variable (M), while the “b” path indicates the association of the mediator with the dependent variable (Y) (the product of these two paths, “a × b”*, constitutes the statistically indirect association). The “c” path represents the total association of the independent variable with the dependent variable (the sum of the direct and indirect associations) and is not displayed as a separate path in Figure 1. Finally, the “c′” path defines the direct association of the independent variable with the dependent variable when the mediator is included in the model.

In the subsequent mediation and moderation models (Table 4 and Figure 1), the DS14 total score was analyzed as a continuous independent variable. The analyses showed that, in the DS14 → HIT-6 model, there were statistically significant indirect associations through HADS-A and VAS, with effect sizes of 0.082 and 0.044, respectively (95% CIs: 0.016–0.157 and 0.012–0.083). In contrast, HADS-D scores did not exhibit a significant indirect association in the relationship between DS14 and HIT-6 (*p* > 0.05). When the DS14 → FAST model was examined, HADS-D was found to have a significant partial mediating role in the relationship between DS14 and FAST (indirect effect = 0.161, 95% CI = 0.043–0.289). However, the indirect associations of HADS-A and VAS with the relationship between DS14 and FAST were not statistically significant (*p* > 0.05) (Table 4).

**Figure 2:**
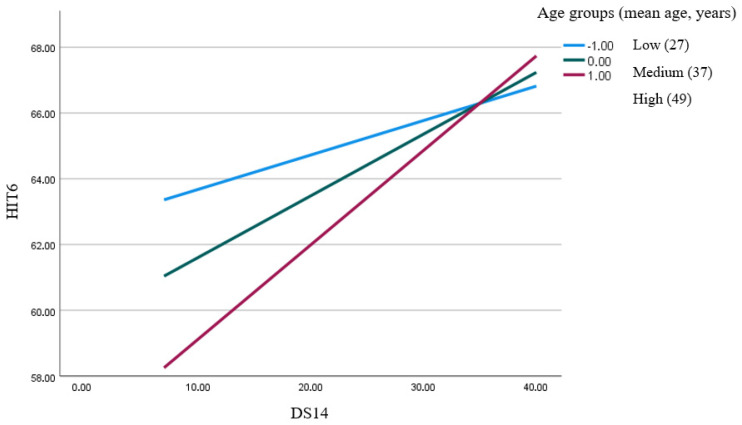
Moderating effect of age on the relationship between Type D personality and HIT-6.

The moderating role of age on the relationship between DS14 and HIT-6 was examined using moderation analysis (Figure 2). The results indicated that the association between DS14 and HIT-6 differed significantly across age groups (DS14 × age interaction: *p* = 0.018, *b* = 0.008, SE = 0.004, 95% CI = 0.001–0.015, R^2^ = 0.158). As shown in Figure 2, the association between increasing DS14 scores and higher HIT-6 scores was more pronounced in older patients compared with those in the younger age group. These findings suggest that the positive association between DS14 scores and HIT-6 scores was stronger at higher ages.

## 4. Discussion

This study examined the relationships between TDP and both headache impact and daily functioning in women diagnosed with migraine, and evaluated the mediating roles of psychological symptoms and pain severity in these associations. According to the findings, women with migraine who exhibited TDP traits showed greater functional impairment in the domains of cognitive functioning, autonomy, financial issues, and interpersonal relationships. In women with migraine, TDP demonstrated moderate to strong positive associations with the impact of headache on daily life, multidimensional functional impairment, and levels of anxiety and depression. Furthermore, statistically significant indirect associations through anxiety symptoms and pain severity were observed in the relationship between TDP and headache impact. Similarly, an indirect association through depressive symptoms was observed in the relationship between TDP and overall functioning. In addition, age emerged as a significant moderator in the association between TDP and headache impact, with this relationship being more pronounced in older patients.

TDP was identified in 41.2% of our sample, a prevalence consistent with findings reported in the existing literature [16]. However, the fact that our sample consisted exclusively of women allowed for the evaluation of the distribution and clinical relevance of TDP traits in women with migraine within a more homogeneous sample. In our study, women with migraine who exhibited TDP traits had lower levels of education and lower employment rates. Similarly, a study conducted with 132 individuals diagnosed with migraine reported lower education levels and employment rates among Type D-positive migraine patients [17]. However, studies addressing this issue, particularly among women with migraine, remain limited in number.

In this study, the prevalence of chronic migraine was found to be significantly higher among Type D-positive patients. In addition, accompanying chronic physical illnesses were observed more frequently in this group. Although studies directly examining this issue in the context of migraine are limited, it is well established that the presence of TDP in chronic diseases and pain conditions is associated with a greater physical and psychological disease burden [33,34]. In our study, the higher prevalence of chronic migraine and physical comorbidities among women with migraine who were Type D-positive suggests that TDP may be a factor that adversely influences the course of migraine and increases the overall disease burden. In contrast to some previous reports, no significant differences were observed between the two groups with respect to mean attack duration or VAS pain scores. In the literature, however, a significant association between the presence of TDP and migraine pain severity has been reported in patients with migraine [16]. This discrepancy may be attributable to the fact that our sample consisted exclusively of women, representing a relatively homogeneous group. Moreover, these findings suggest that TDP may exert its influence not only through objective pain parameters but also through subjective experiences and perceived disease burden.

In this study, multidimensional associations were identified between TDP and both psychosocial and clinical variables in women with migraine. As TDP traits increased, greater impairment was observed in overall functioning as well as across all functional subdomains in women with migraine. Several studies have reported that the presence of TDP in individuals with migraine is associated with greater pain severity, higher functional impairment scores, and markedly poorer quality of life [16,17,35]. In our sample, higher levels of TDP traits were significantly and positively correlated with impairments across multiple functional domains, including occupational functioning, autonomy, cognitive functioning, financial issues, interpersonal functioning, and leisure activities. These findings suggest that, in women with migraine, TDP may be associated with an inability to maintain an autonomous lifestyle, achieve adequate occupational performance, and effectively utilize cognitive abilities, potentially due to heightened stress levels. Indeed, one possible consequence of these functional impairments is the lower educational attainment and employment rates observed in the Type D+ group in our study. Moreover, heightened stress and anxiety may contribute to difficulties in interpersonal relationships and limit engagement in enjoyable leisure activities due to symptom burden. TDP has been shown to substantially affect various domains of functioning not only in the general population but also across a range of chronic medical conditions [36,37,38,39]. However, studies that examine the relationship between migraine and such a broad range of functional subdomains, particularly those focusing specifically on female samples, are quite limited.

One of the important findings of our study is that, in women with migraine, anxiety and depression levels showed significant positive correlations with both the impact of headache on daily life and overall functioning. In the literature, while several studies have demonstrated that anxiety and depression substantially affect headache-related daily activities in individuals with migraine [40,41], other studies have reported no such association [42]. In our study, however, anxiety and depression levels in women with migraine were found to show significant and consistent associations with both the impact of headache on daily life and multiple domains of overall functioning.

In our study, when control variables such as anxiety and depression were included in the analyses, the between-group difference in headache impact was no longer statistically significant. This finding may be considered consistent with the existing literature. Previous research has demonstrated that psychological factors accompanying migraine are associated with greater headache-related impact on daily life [43,44]. This suggests that the relationship between TDP and the impact of headache on daily life may not be direct, but rather influenced by accompanying psychological factors. Indeed, in our study, anxiety levels and pain severity showed significant indirect associations with the relationship between TDP and the impact of headache on daily life in women with migraine. These findings suggest that anxiety symptoms and perceived pain severity may be involved in the observed association between TDP and headache impact in women with migraine. However, because all variables were measured at the same time point, the directionality of these relationships cannot be determined and the findings should be interpreted as exploratory indirect statistical associations rather than temporal pathways. In the literature, it has been shown that anxiety can mediate the relationship between TDP and quality of life and functioning in certain medical conditions [45,46]. In another study, TDP and migraine attack severity were shown to have a significant and strong association with migraine-related quality of life [16]. Interestingly, while the association between Type D personality and headache impact became non-significant after adjustment for anxiety and depression, the associations between Type D personality and overall functioning remained significant across multiple domains. This finding is consistent with the exploratory mediation analyses and suggests that concurrent psychological symptoms may account for a substantial proportion of the observed association between Type D personality and headache impact, whereas the association with functional impairment appears to be more robust and less dependent on anxiety and depressive symptom levels.

Another noteworthy finding of our study is that an indirect association through depressive symptoms was observed in the relationship between TDP and overall functioning in women with migraine. Individuals with TDP traits frequently exhibit negative affectivity, social inhibition, and feelings of helplessness, a pattern that has been associated with increased vulnerability to depressive symptoms [47]. Depressive symptoms are known to be associated with reduced motivation, diminished interest, and low energy levels, all of which may be related to poorer functioning across multiple domains. However, the cross-sectional nature of the present study does not allow conclusions regarding the temporal ordering of these associations [48]. In a study conducted among patients with migraine, TDP and depression were identified as determinants of quality of life, with the model explaining approximately 53% of the variance [17]. Our findings suggest that depressive symptoms may represent an important correlate of the observed association between TDP traits and functional impairment in women with migraine.

In our study, the relationship between TDP and the impact of headache on daily life was observed to be stronger in older women with migraine. This finding may be related to increased vulnerability with advancing age, reduced physical resilience, diminished psychological flexibility, or more limited coping strategies [17,49,50]. On the other hand, it is well established that fluctuations in estrogen levels play an important role in the frequency and severity of migraine attacks [8]. One possible explanation is that psychological and coping-related factors may become increasingly relevant in the perception and management of migraine-related burden with advancing age. However, this interpretation remains speculative and warrants investigation in future studies.

In light of these findings, our study demonstrates that TDP traits in women with migraine are closely associated with the clinical characteristics of the disorder, psychological factors, the impact of headache on daily life, and multidimensional functional impairment. Furthermore, the study elucidates the multifaceted relationships between TDP and clinical outcomes of migraine through factors such as age, anxiety, depression, and pain severity. These results underscore the importance of psychological assessment and follow-up in women with migraine who exhibit TDP traits, as such approaches may be critical for preserving functional levels alongside effective headache management.

### Limitations

The cross-sectional design of the study precludes the establishment of causal relationships. In addition, the sample was drawn from a single center and consisted exclusively of women, which limits the generalizability of the findings. These indirect associations should be interpreted as exploratory, as the cross-sectional design does not permit conclusions regarding temporal or causal pathways. Moreover, the predominantly self-report nature of the assessment instruments introduces the potential risk of response bias. Although the presence of comorbidities was controlled for, the effects of other clinical conditions accompanying migraine on psychosocial variables could not be fully disentangled. Due to the large number of group comparisons, correlations, and subgroup analyses performed without adjustment for multiple testing, there is an increased risk of Type I error. Therefore, these findings should be interpreted as exploratory and require replication in future studies.

## 5. Conclusions

In this study of women with migraine, Type D personality was significantly associated with multidimensional functional impairment. Although patients with Type D personality reported greater headache impact, this association was no longer statistically significant after adjustment for clinical and psychological factors. Indirect associations involving anxiety symptoms, depressive symptoms, and pain severity suggest that psychological factors may play an important role in the relationship between Type D personality and migraine-related outcomes. In addition, psychological variables such as anxiety, depression, and pain severity showed significant indirect associations with these relationships, while age emerged as a moderating variable in the association between TDP and headache impact. When all findings are considered together, it may be inferred that managing migraine alone may be insufficient in a disorder that substantially impairs functioning. Incorporating psychological factors and personality characteristics into disease management may contribute to both more effective headache control and the preservation of overall functioning. Therefore, further research, particularly longitudinal studies designed to elucidate the temporal and functional relationships between personality structure and headache, is warranted.

To our knowledge, this is the first study specifically conducted in women with migraine to simultaneously examine Type D personality, headache impact, multidimensional functioning, and indirect statistical associations involving anxiety, depression, pain severity, and age.

## Figures and Tables

**Table 1:** Comparison of sociodemographic characteristics according to the presence of Type D personality in women with migraine.

Mean ± Standard Deviation, *n* (%)	Type D− (*n* = 120)	Type D+ (*n* = 84)	Statistic	*p*
**Age**	37.78 ± 10.48	39.10 ± 10.37	t = −0.884	0.378
**Education level**	12.57 ± 3.95	10.75 ± 4.56	t = 2.959	**0.004**
**Marital status**			X^2^ = 0.474	0.491
Married	85 (70.8%)	64 (76.2%)		
Single	35 (29.2%)	20 (23.8%)		
**Employment status**			X^2^ = 12.007	**<0.001**
Employed	71 (59.2%)	29 (34.5%)		
Unemployed	49 (40.8%)	55 (65.5%)		
**Place of residence**			X^2^ = 1.716	0.190
Urban	112 (93.3%)	73 (86.9%)		
Rural	8 (6.7%)	11 (13.1%)		
**Smoking**			X^2^ = 0.906	0.341
Yes	38 (31.7%)	32 (38.1%)		
No	82 (68.3%)	52 (61.9%)		
**Alcohol use**			X^2^ = 3.190	0.074
Yes	18 (15%)	5 (6%)		
No	102 (85%)	79 (94%)		
**Physical activity**			X^2^ = 0.389	0.533
Yes	33 (27.5%)	19 (22.6%)		
No	87 (72.5%)	65 (77.4%)		

*n*: number of observations, *p*: level of statistical significance, t: *t*-test statistic, X^2^: chi-square test statistic. Statistically significant *p*-values are presented in bold (*p* < 0.05).

**Table 2:** Comparison of clinical characteristics, psychiatric symptoms, and functioning levels according to the presence of Type D personality in women with migraine.

Variable	Type D− (*n* = 120)	Type D+ (*n* = 84)	Statistic	*p*
**Migraine type**			X^2^ = 5.627	**0.018**
Episodic	95 (79.2%)	53 (63.1%)		
Chronic	25 (20.8%)	31 (36.9%)		
**Psychiatric history**			X^2^ = 1.264	0.261
Yes	38 (31.7%)	33 (39.3%)		
No	82 (68.3%)	51 (60.7%)		
**Chronic medical comorbidity**			X^2^ = 4.573	**0.032**
Yes	29 (24.2%)	32 (38.1%)		
No	91 (75.8%)	52 (61.9%)		
**Migraine duration (years)**	8.5 (3–14.5)	6 (3–10)	Z = −1.261	0.207
**Migraine attack duration (min)**	360 (195–1440)	360 (180–1440)	Z = −0.840	0.401
**Mean VAS score**	8 (7–10)	9 (7–10)	Z = −1.639	0.101
**Number of headache days per month**	4 (2–8)	5 (3–15)	Z = −2.467	**0.014**
**HADS** **–** **A**	6.76 ± 4.09	11.79 ± 3.60	t = −9.080	**<0.001**
**HADS** **–** **D**	5.48 ± 3.91	10.19 ± 3.89	t = −8.470	**<0.001**
**HIT** **–** **6**	62.66 ± 8.45	66.31 ± 6.03	F = 1.886	0.171*
**FAST (total)**	20.38 ± 12.27	38.46 ± 14.41	F = 7.977	0.005 *
Autonomy	3.42 ± 3.41	6.77 ± 2.98	F = 7.023	0.009 *
Occupational functioning	4.23 ± 4.76	7.87 ± 4.05	F = 0.252	0.616*
Cognitive functioning	4.91 ± 3.99	8.56 ± 3.51	F = 8.409	0.004 *
Financial issues	1.27 ± 1.95	2.70 ± 2.10	F = 4.768	0.030 *
Interpersonal relationships	4.43 ± 4.45	9.26 ± 4.24	F = 12.911	<0.001 *
Leisure activities	2.12 ± 1.92	3.30 ± 1.71	F = 1.701	0.194 *

*n*: number of observations, *p*: level of statistical significance, Q1: first quartile, Q3: third quartile, F: MANCOVA test statistic, t: *t*-test statistic, X^2^: chi-square test statistic, Z: Mann–Whitney U test statistic. Continuous variables are presented as mean ± standard deviation or median (Q1–Q3), and categorical variables as n (%). VAS: Visual Analog Scale, HADS: Hospital Anxiety and Depression Scale, HIT−6: Headache Impact Test–6, FAST: Functioning Assessment Short Test. * Controlled for education level, employment status, migraine type, presence of chronic medical comorbidity, number of headache days per month, and anxiety and depression scores. Statistically significant *p*-values are presented in bold (*p* < 0.05).

**Table 3:** Correlations between Type D personality and clinical and psychosocial variables (*n* = 204).

		HIT-6	FAST	Autonomy	Occupational Functioning	Cognitive Functioning	Financial Issues	Interpersonal Relationships	Leisure Activities	HADS-A	HADS-D	VAS	Number of Headache Days per Month	Migraine Duration (Years)	Migraine Attack Duration (Minutes)
DS14	r: *p*:	**0.346** **<0.001**	**0.625** **<0.001**	**0.560** **<0.001**	**0.546** **<0.001**	**0.539** **<0.001**	**0.407** **<0.001**	**0.607** **<0.001**	**0.368** **<0.001**	**0.708** **<0.001**	**0.653** **<0.001**	**0.193** **0.006**	**0.177** **0.011**	−0.047 0.500	0.016 0.816
Negative affectivity	r: *p*:	**0.332** **<0.001**	**0.601** **<0.001**	**0.531** **<0.001**	**0.564** **<0.001**	**0.507** **<0.001**	**0.374** **<0.001**	**0.583** **<0.001**	**0.320** **<0.001**	**0.735** **<0.001**	**0.639** **<0.001**	**0.180** **0.010**	**0.183** **0.009**	−0.019 0.784	0.029 0.683
Social inhibition	r: *p*:	**0.288** **<0.001**	**0.519** **<0.001**	**0.475** **<0.001**	**0.408** **<0.001**	**0.461** **<0.001**	**0.360** **<0.001**	**0.506** **<0.001**	**0.345** **<0.001**	**0.525** **<0.001**	**0.534** **<0.001**	**0.168** **0.017**	0.132 0.060	−0.077 0.274	0.012 0.864
VAS	r: *p*:	**0.456** **<0.001**	**0.202** **0.004**	**0.208** **0.003**	**0.239** **<0.001**	**0.166** **0.018**	0.107 0.126	0.118 0.092	**0.161** **0.021**	**0.235** **<0.001**	0.119 0.090				
Number of headache days per month	r: *p*:	**0.170** **0.015**	**0.229** **0.001**	**0.175** **0.012**	**0.194** **0.005**	**0.204** **0.003**	**0.180** **0.010**	**0.227** **0.001**	**0.144** **0.040**	**0.138** **0.040**	**0.138** **0.050**				
Migraine duration (years)	r: *p*:	**0.145** **0.038**	−0.006 0.932	0.007 0.919	0.004 0.955	0.001 0.994	−0.050 0.481	−0.063 0.368	0.129 0.065	0.096 0.172	0.034 0.628				
Migraine attack duration (minutes)	r: *p*:	**0.177** **0.011**	0.034 0.628	0.067 0.338	0.091 0.195	−0.038 0.592	−0.038 0.592	−0.014 0.842	−0.013 0.851	0.024 0.729	−0.011 0.877				
HADS-A	r: *p*	**0.348** **<0.001**	**0.509** **<0.001**	**0.478** **<0.001**	**0.511** **<0.001**	**0.378** **<0.001**	**0.360** **<0.001**	**0.428** **<0.001**	**0.365** **<0.001**						
HADS-D	r: *p*	**0.298** **<0.001**	**0.514** **<0.001**	**0.507** **<0.001**	**0.452** **<0.001**	**0.444** **<0.001**	**0.339** **<0.001**	**0.462** **<0.001**	**0.303** **<0.001**						

r: correlation coefficient, *p*: level of statistical significance. Statistically significant *p*-values are presented in bold (*p* < 0.05). DS14: Type D Personality Scale, VAS: Visual Analog Scale, FAST: Functioning Assessment Short Test, HADS: Hospital Anxiety and Depression Scale, HIT-6: Headache Impact Test–6.

**Table 4:** Results of exploratory mediation analyses examining indirect statistical associations involving HADS-A, HADS-D, and VAS (*n* = 204).

X	Y	M	Path	*b*	SE	*p*	95% CI	R^2^	K^2^
**DS14**	**HIT–6**	**HADS** **–** **A**	a	0.237	0.017	**<0.001**	0.204–0.270	0.502	-
			b	0.348	0.156	**0.027**	0.041–0.055	0.141	-
			c′	0.112	0.052	**0.033**	0.009–0.215	0.141	-
			c	0.194	0.037	**<0.001**	0.121–0.268	0.119	-
			**Indirect effect**	0.082	0.036	**-**	0.017–0.157	-	0.147
		**HADS** **–** **D**	a	0.215	0.018	**<0.001**	0.180–0.249	0.427	-
			b	0.217	0.149	0.147	−0.077–0.511	0.129	-
			c′	0.148	0.049	**0.003**	0.051–0.245	0.129	-
			c	0.194	0.037	**<0.001**	0.121–0.268	0.119	-
			**Indirect effect**	0.047	0.028	-	−0.009–0.103	-	0.083
		**VAS**	a	0.024	0.009	**0.006**	0.007–0.041	0.037	-
			b	1.837	0.278	**<0.001**	1.289–2.384	0.277	-
			c′	0.151	0.034	**<0.001**	0.083–0.218	0.277	-
			c	0.194	0.037	**<0.001**	0.121–0.268	0.119	-
			**Indirect effect**	0.044	0.017	**-**	0.012–0.080	-	0.078
	**FAST**	**HADS** **–** **A**	a	0.237	0.017	**<0.001**	0.204–0.270	0.502	-
			b	0.532	0.310	0.087	−0.079–1.143	0.399	-
			c′	0.710	0.104	**<0.001**	0.506–0.915	0.399	-
			c	0.837	0.074	**<0.001**	0.692–0.981	0.391	-
			**Indirect effect**	0.126	0.073	-	−0.012–0.271	-	0.094
		**HADS** **–** **D**	a	0.215	0.018	**<0.001**	0.180–0.249	0.427	-
			b	0.751	0.292	**0.011**	0.175–1.327	0.410	-
			c′	0.675	0.096	**<0.001**	0.486–0.865	0.410	-
			c	0.837	0.074	**<0.001**	0.691–0.982	0.391	-
			**Indirect effect**	0.161	0.063	**-**	0.043–0.293	-	0.120
		**VAS**	a	0.024	0.009	**0.006**	0.007–0.041	0.037	-
			b	0.918	0.604	0.130	−0.272–2.108	0.398	-
			c′	0.815	0.075	**<0.001**	0.667–0.962	0.398	-
			c	0.837	0.074	**<0.001**	0.692–0.982	0.391	-
			**Indirect effect**	0.022	0.017	-	−0.006–0.061	-	0.016

X: independent variable, Y: dependent variable, M: mediator, a: path from X to M, b: path from M to Y, c′: direct path from X to Y, c: total path from X to Y, *b*: unstandardized beta coefficient, SE: standard error, *p*: statistical significance level, CI: confidence interval, R^2^: coefficient of determination, K^2^: effect size. DS14: Type D Personality Scale, VAS: Visual Analog Scale, HADS: Hospital Anxiety and Depression Scale, HIT–6: Headache Impact Test–6, FAST: Functioning Assessment Short Test. Statistically significant *p*-values are presented in bold (*p* < 0.05). Since paths b and c′ are in the same regression model, the same R^2^ values were reported.

## Data Availability

Data are available from the corresponding author upon reasonable request.

## Abbreviations

The following abbreviations are used in this manuscript:

DS14	Type D Personality Scale
VAS	VAS: Visual Analogue Scale
HADS	Hospital Anxiety and Depression Scale
HADS-A	Hospital Anxiety and Depression Scale—Anxiety subscale
HADS-D	Hospital Anxiety and Depression Scale—Depression subscale
HIT-6	Headache Impact Test—6
FAST	Functioning Assessment Short Test
ICHD-3	International Classification of Headache Disorders, 3rd Edition
MANCOVA	Multivariate Analysis of Covariance
PROCESS	Hayes’ PROCESS Macro for SPSS
CI	Confidence Interval
K^2^	Kappa-squared (Effect size indicator in mediation analysis)
